# Step-by-step *En face* O red oil method for aortic plaque staining and quantification in ApoE knockout mouse

**DOI:** 10.1016/j.mex.2026.104020

**Published:** 2026-06-27

**Authors:** Nurin Yasmin Mohd Khairudin, B. Vimala R.M.T Balasubramaniam, Suhaila Abd Muid, Nurdiyana Nasrudin, Nasibah Azme

**Affiliations:** a Laboratory Animal Care Unit (LACU), Faculty of Medicine, Universiti Teknologi MARA, Sungai Buloh, Selangor, Malaysia; b Institute of Medical Molecular Biotechnology, Faculty of Medicine, Universiti Teknologi MARA, Sungai Buloh, Selangor, Malaysia; c Nutrition, Metabolism & Cardiovascular Research Centre, Institute for Medical Research, Selangor, Malaysia; d Department of Biochemistry and Molecular Medicine, Faculty of Medicine, Universiti Teknologi MARA, Sungai Buloh, Selangor, Malaysia; e Department of Pediatrics, Faculty of Medicine, Universiti Teknologi MARA, Sungai Buloh, Selangor, Malaysia; f Department of Physiology, Faculty of Medicine, Universiti Teknologi MARA, Sungai Buloh, Selangor, Malaysia; g Department of Medical Education, Faculty of Medicine, Universiti Teknologi MARA, Sungai Buloh, Selangor, Malaysia

**Keywords:** Oil red O, *en face*, Step-by-step, Atherosclerosis

## Abstract

The *en face* Oil Red O (ORO) technique is an established method for quantifying experimental atherosclerotic plaque. However, interlaboratory reproducibility is influenced by procedural factors, including aorta dissection, image preparation, and analysis. This paper provides a detailed step-by-step procedure for *en face* ORO analysis of ApoE knockout mice, with comprehensive photographic guidance for each step, for plaque staining and quantification using a standard digital camera and Adobe Photoshop. The procedure includes harvesting and trimming the aorta, staining, mounting, imaging, and quantitative analysis. Images are captured using generic digital cameras and plaques are quantified in Adobe Photoshop. Each step is documented photographically, providing a visual reference to aid protocol implementation and enhance methodological transparency. The workflow offers a practical and reproducible method for standardized *en face* plaque evaluation and could be valuable for novice researchers performing preclinical atherosclerosis studies.

This method:

• Provides a standardized, photographically documented workflow for *en face* ORO staining and plaque quantification in ApoE knockout mice.

• Enables accurate plaque measurement using a conventional digital camera and Adobe Photoshop.

• Includes practical photo guidance from aorta dissection through image capture and lesion measurement.

## Graphical abstract

**Figure:**
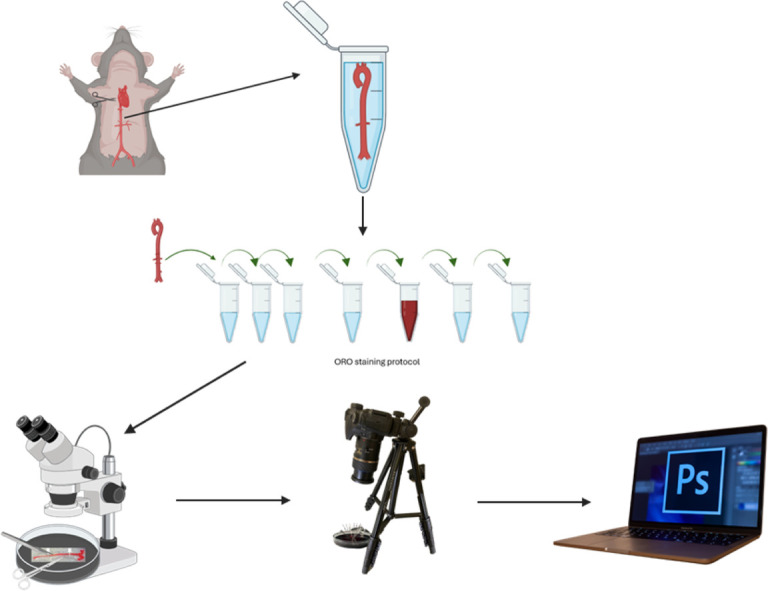

## Specifications table

**Table:** 

**Subject area**	Medicine and Dentistry
**More specific subject area**	*En face* aortic plaque staining and quantification
**Name of your method**	Step-by-step *en face* ORO staining and Adobe Photoshop-based plaque quantification for aortic plaque in ApoE knockout mouse
**Name and reference of original method**	*En face* ORO staining protocol: Beattie et al. 2009 [1], Lin et al. 2015 [2] Photoshop-based image analysis: Lehr et al. 1997 [3], Lehr et al. 1999 [4], Torzewski et al. 2008 [5]
**Resource availability**	N/A

## Background

Atherosclerosis is a chronic inflammatory disease of large arteries that is characterized by the formation of lipid-rich plaques in the arterial wall. This continuous process underpins most of the cardiovascular diseases (CVDs) and strokes, and thus, it is one of the main contributors to morbidity and mortality in the global context.Apolipoprotein E (ApoE) knockout mice, in particular, are widely used as experimental murine models to study the mechanisms of plaque formation as well as to determine the effectiveness of therapeutic interventions [1,6].

The method of Oil Red O (ORO)-stained-lesion quantification is essential for accurately measuring the atherosclerotic burden, especially in regions such as the aortic root and its branches, where lesions typically develop early [7]. Classic cross-sectional histology of the aortic root or aorta can still be used to localize lesions, but its labour-intensive nature and inability to provide a complete spatial profile of plaque distribution are major limitations. Conversely, *en-face* ORO staining permits the study of the arterial surface as a whole, thereby enabling a holistic depiction of plaque distribution throughout the entire aorta, starting from the aortic arch and the branching vessels down to the iliac aorta. The *en face* orientation allows whole-mount examination of the arterial surface and provides a complete picture of plaque distribution [8]. Due to the possibility of atherosclerotic lesions forming at any point of the aortic tree, the whole-aorta *en-face* ORO staining method has a unique advantage of assessing lipid-containing plaques in the entire aorta and its branches in the same mouse [9].

There are a number of established *en face* ORO procedures described and methodological studies reported in the literature addressing various aspects of aortic dissection, staining, imaging, and plaque quantification [1,2,[8], [9], [10], [11], [12], [13]]. These studies have contributed substantially to the standardization of en face plaque assessment in experimental atherosclerosis. There are, however, some variations in dissection methods, staining protocols, image acquisition, and plaque quantification methods. Moreover, there is significant variation in the extent of visual guidance and method documentation in these studies. Although representative images, schematic illustrations, or image-analysis workflows are provided by some studies, comprehensive photographic documentation of the entire workflow, from aortic harvesting and preparation to staining, imaging, and quantitative analysis, is not consistently reported throughout the literature.

In addition to these already published methodologies, the present study offers a detailed step-by-step *en face* ORO staining and quantification procedure with extensive photographic guidance. The workflow is clear and easy to follow, and a series of photographs is included at each step of the procedure, from aortic excision and trimming to staining, mounting, acquisition, and quantitative analysis. The quantification of plaque is illustrated with a standard digital camera and Adobe Photoshop software, and screenshot instructions are provided for the purposes of image processing and measurement. This protocol combines detailed visual guidance with quantitative image-analysis procedures, with the aim of increasing methodological transparency, improving reproducibility, and facilitating the wider use of standardized *en face* plaque assessment, especially among novice researchers and laboratories seeking a practical, accessible method to evaluate *en face* plaques. Table 1 compares the methodological characteristics of previously published studies with the current study.

**Table 1:** Comparison of methodological features reported in previously published *en face* ORO staining protocols and the present study.

Study	Detailed Dissection Protocol	Step-By-Step ORO Staining	Descriptive Protocol Figures	Instrument Setup Illustrated	Quantification Method Described	Quantification Workflow Illustrated
Beattie et al., 2009 [1]	Partial	Yes	No	No	Yes: ORO extraction/ spectrophotometry	No
Lin et al., 2015 [2]	Yes	Yes	Partial	No	Yes: WinROOF	No
Chen et al., 2022 [8]	Partial	No	Partial	No	Partial	No
Chen et al., 2023 [9]	Yes	Yes	Yes	Partial	Yes: ImageJ	Yes
Liu & Xu, 2025 [10]	Yes	Yes	Yes	Partial	Yes: ImageJ	Yes
Mohanta et al., 2016 [11]	Yes	Yes	Partial	Yes	Yes: ImageJ	Partial
Andrés-Manzano et al., 2015 [12]	Yes	Yes	Partial	Partial	Partial: SigmaScan Pro	No
Chan et al., 2022 [13]	Yes	Yes	Partial	No	Yes: ImageJ	Partial
**Present Study**	**Yes**	**Yes**	**Yes**	**Yes**	**Yes (Adobe Photoshop)**	**Yes**

Yes = feature comprehensively described or illustrated; Partial = feature mentioned, briefly described, or represented by selected images/schematics without comprehensive visual guidance; No = feature not reported or not illustrated.

Table 1 summarizes the contributions of previous protocols to the standardization of *en face* plaque assessment. The present protocol complements these methodologies by combining extensive photographic documentation and detailed instrument instructions with plaque quantification via screen-guided Adobe Photoshop software throughout aortic harvesting, tissue preparation, staining, image acquisition, and quantitative analysis.

## Method details

### Harvesting of the aorta from the aortic root to the iliac

**Fig. 1:**
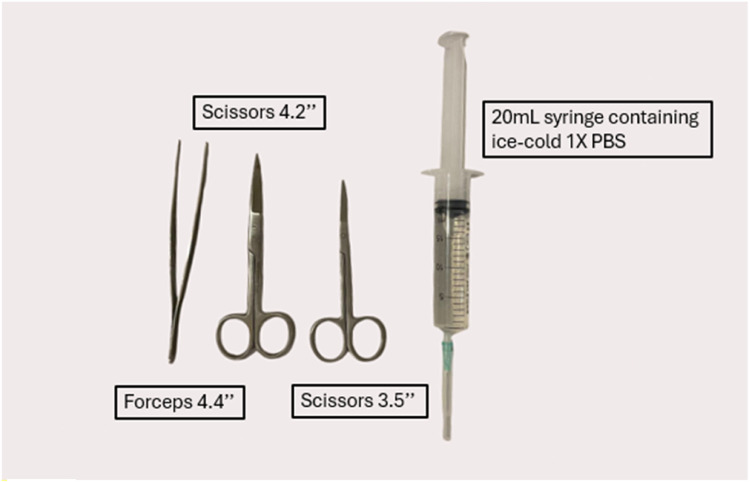
Setup for gross dissection of anatomical strucures prior to microscopic dissection of the aorta.

In this experiment, an ApoE knockout mouse obtained from Taconic Bioscience, USA was utilized. The ApoE knockout mouse used in this protocol is 70 weeks old, having been maintained on a normal diet since week 0. Before anesthesia, the materials needed were assembled as indicated in Fig. 1.

**Fig. 2:**
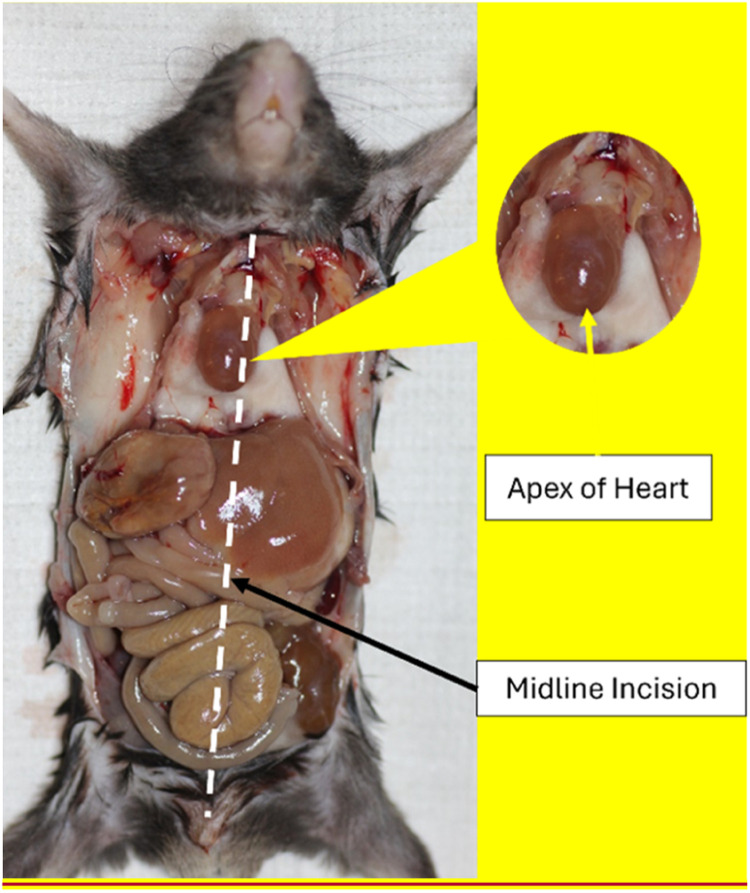
Midline incision performed on mice to expose the organ and an enlarged view of the apex of the heart for 1X PBS perfusion purpose.

To administer the anesthesia, the mouse was placed in a supine position. The mouse was administered an overdose of anesthesia (100mg/mL ketamine and 20mg/mL xylazine cocktail) at 0.01 mL per 10 g of body weight using a 1 mL syringe (TERUMO Syringe 1 mL Luer Slip) and a 30 G needle (TERUMO Agani Needle 30 G x 1/2′). Once the anesthesia has registered, a midline incision was performed using a pair of scissors, extending cranially from the abdominal region to the thoracic area to expose internal organs for dissection as shown in Figure 2 using 4.8’ scissors and 4.7’ forceps. Prior to evisceration of thoracic and abdominal cavities, 20 mL of ice-cold 1X PBS was used to perfuse the circulatory system connected to the aorta, including the organs, by pushing the 1X PBS through the apex of the heart as in Fig. 2 using a 20 mL syringe (Hospitech Disposable Syringe 20 mL Leur Lock) and 23 G needle (TERUMO Agani Needle 23 G x 1’).

The perfusion was carried out as a precaution to ensure that blood and clot were removed for clear visualization, to prevent accelerated tissue degradation caused by blood residue, to ensure even staining later, since ORO stains the plaque a red color, and finally to improve accuracy in quantitative analysis.

**Fig. 3:**
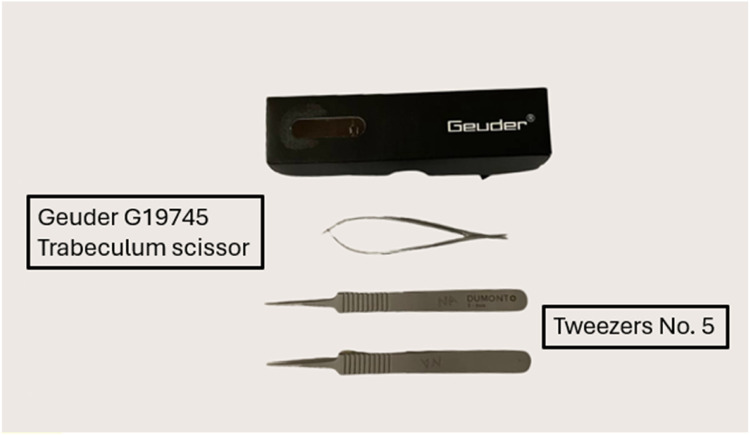
Microdissection setup used to remove adventitial fat from the aorta, allowing for clear visualization of its overall morphology.

Once the organs are exposed, the abdominal cavity is emptied first, followed by excision and removal of the lungs to facilitate the examination of the thoracic cavity. After the gross dissection was completed, the mouse was transferred to perform microscopic dissection under a stereo zoom microscope (AmScope 3.5X–180X Manufacturing 144-LED Zoom). The dissecting instruments needed for this procedure are shown in Fig. 3.

**Fig. 4:**
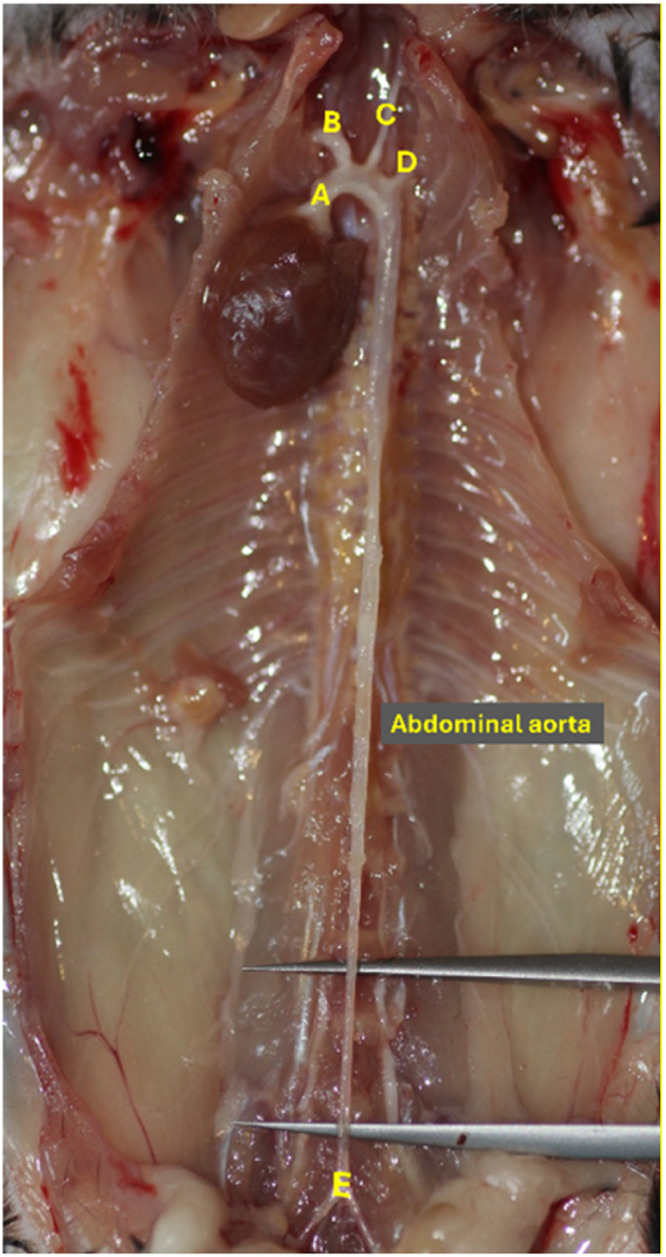
Exposed aorta labeled with A-E to indicate incision points to detach the aorta from the spine. A- ascending aorta; B- brachiocephalic trunk; C-left common carotid artery; D- left subclavian artery; E- iliac artery.

Next, the adventitial lipid layers were removed meticulously to expose the aortic branch using fine-stitch scissor (Geuder G19745) and fine tweezers (Dumont Style 2 Inox 5 tweezers). Then, the adventitial lipid layers originate from the aortic arch (brachiocephalic trunk, left common carotid artery, and left subclavian artery) and extend to the ascending aorta, before descending towards the thoracic, abdominal, and iliac branches, which were inspected and removed. Once the whole aorta was devoid of the adventitial fat, the aorta was isolated from the spine by making vertical incisions at the ascending aorta, brachiocephalic trunk, left common carotid artery, left subclavian artery, and common iliac artery to detach the whole length of the aorta from the spine as indicated in Fig. 4.

### Implementation of the *en face* technique in aortic tissue

**Fig. 5:**
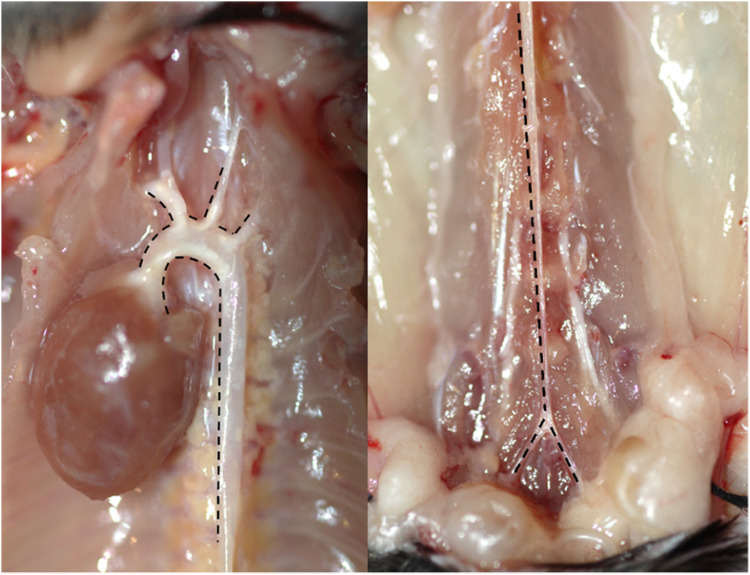
Dashed line indicates the incision made throughout the whole aorta using fine stitch scissor to expose the tunica intima, thus creating the *en face* orientation.

Prior to ORO staining, the aorta was incubated in 10% neutral buffered formalin for 48 h. After incubation, the tunica intima was exposed using the *en face* method by making incisions as shown in Fig. 5 using a fine-stitch scissor (Geuder G19745) and tweezers (Dumont Style 2 Inox 5 tweezers) under a microscope (AmScope 3.5X–180X Manufacturing 144-LED Zoom).

The incisions on the aorta were made by laying the aorta flat on a black Sylgaard Petri Dish (DMT) and pinning it to the petri dish using 0.10 mm ultra-thin micro-headless pins (Entosupplies) to assist in fixing the aorta during the incision process. Additionally, sterile water was applied dropwise as needed using a 1 mL syringe (TERUMO Luer Slip syringe) to prevent the aorta from drying, which may cause the tissue to break.

### Staining of *en face* aortic tissue using ORO

**Fig. 6:**
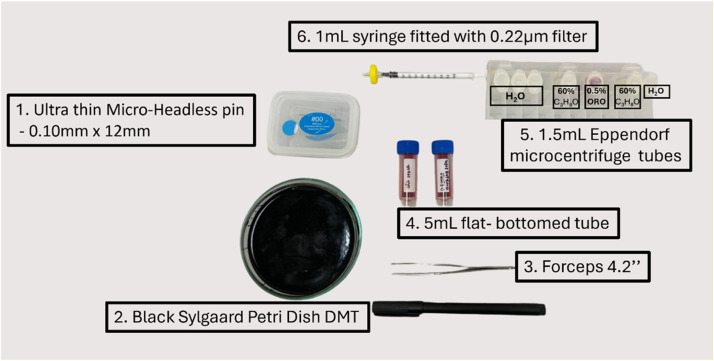
Instrument used for ORO staining procedure; 1,2- To pin the aorta on Black Sylgaard Petri Dish DMT; 3- To transfer the aorta from one staining solution to another; 4- To prepare 0.5% ORO working solution; 5- To immerse the aorta in different staining solutions; 6- To filter 0.5% ORO working solution prior to using in staining.

Prior to staining, the setup was assembled as indicated in Fig. 6.

**Fig. 7:**
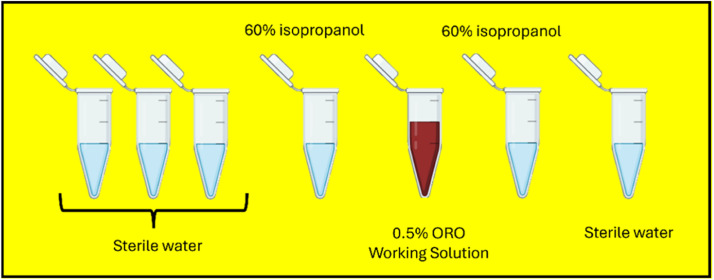
Solutions used for ORO staining of the aorta.

ORO staining procedures were performed based on a modified protocol from previous studies, combining elements from the protocols of Beattie et al. [1] and Lin et al.[2]. ORO working solution was first prepared by mixing three parts of ORO-stock solution (Sigma Aldrich, USA) and two parts of water to obtain a 0.5% ORO-isopropanol solution that was diluted (vol/vol). The working solution was then filtered using a 0.22-micron nylon syringe filter fitted to a 1 mL syringe. It should be filtered slowly and carefully to avoid back splash and ensure the impurities are filtered thoroughly. Then, the following solutions will be prepared for *en face* ORO staining: 0.5% ORO working solution, 60% isopropanol, and sterilized water, in 1.5 mL microcentrifuge tubes (Fig. 7). Forcep is used to transfer the aorta from one staining solution to another.

**Fig. 8:**
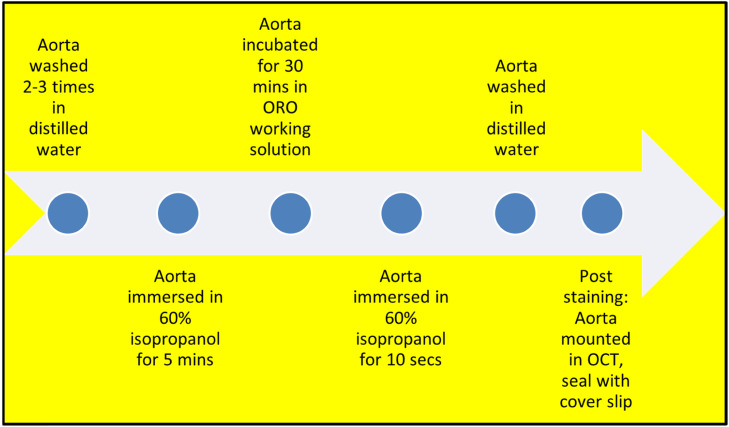
Stepwise workflow of aortic ORO staining, illustrating the sequence of solutions used and corresponding incubation times.

The aorta was then incubated in a detailed sequence according to the respective incubation time (Fig. 8). The incubation time in this current study was modified from a previous study, combining elements from the protocols of Beattie et al. [1] and Lin et al. [2].

### Imaging of *en face* aortic tissue using AmScope stereomicroscope

**Fig. 9:**
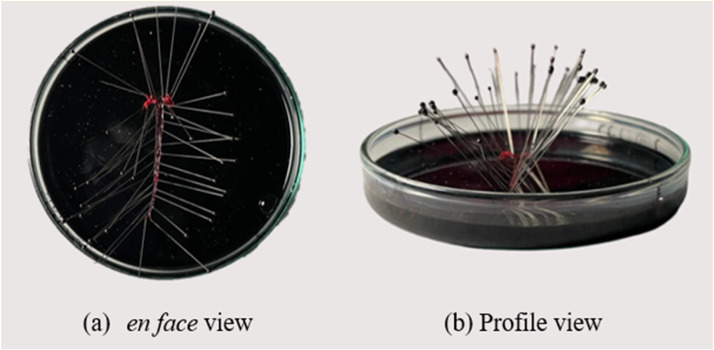
Aorta pinned on a black Sylgaard Petri Dish; (a) *en face* view to visualize the plaque area during quantification; (b) profile view showing ultra-thin pins angled to maintain a clear view of the aorta.

After staining the aorta with ORO solution, it was re-pinned on a black Sylgaard Petri Dish (DMT) (Fig. 9) for viewing under the stereomicroscope (AmScope 3.5X–180X Manufacturing 144-LED Zoom, USA).

**Fig. 10:**
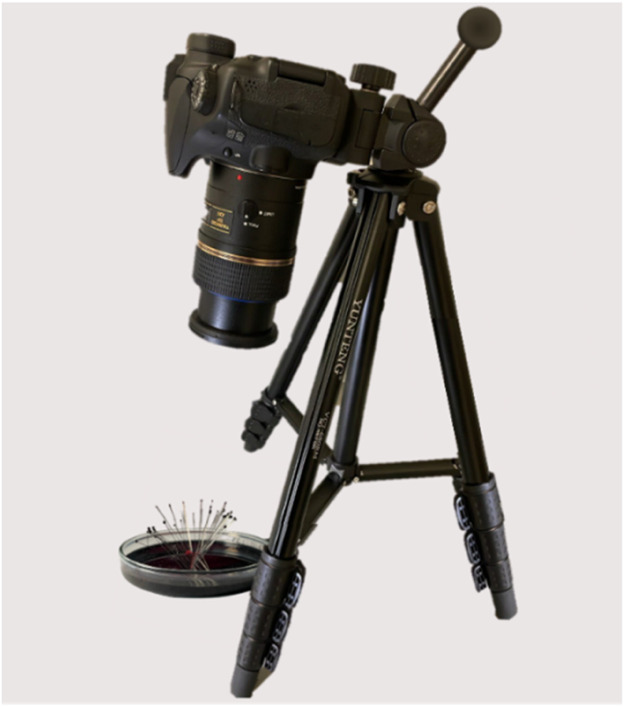
Pinned aorta mounted on a Black Sylgard Petri dish (DMT) and photographed using a Canon EOS 50D (Canon, Tokyo, Japan).

To capture the visualization of the red-stained plaque in the aorta, a Canon EOS 50D (Canon, Tokyo, Japan) was utilized. The digital camera was mounted on a digital stand to facilitate focal length adjustments. Then, the black Sylgaard Petri dish (DMT) was placed under the lens at a position that allowed full view of the aorta (Fig. 10).

A few images were captured of one aorta, with minor or major light adjustments made to ensure the best possible visualization of red-stained plaque throughout the aorta. Once image acquisition was completed, the most representative image for each aorta was carefully selected for quantitative analysis using Adobe Photoshop.

### Quantification of plaque lesion via adobe photoshop

The quantification process was based on previously developed Adobe Photoshop-based image-analysis tools [[3], [4], [5]] and integrated into the existing ORO en-face workflow, which enables measurement of the plaque using standard software of Adobe Photoshop. Using Adobe Photoshop (Version 26.10), ORO-stained regions were outlined through the Polygonal Lasso tool, and the number of pixels within the selected region was determined through the Histogram tool. The percentage of plaque coverage was calculated as following this formula: Total Percentage of Plaque Area = Area of Artery containing plaque / Total Area of artery x 100%.

The following are the step-by-step procedures consisting of Steps 1–16 to obtain the data for use in the formula above using Adobe Photoshop:

1. Open the Adobe Photoshop app.
2. Go to File, Click Open (Fig. 11).

**Fig. 11:**
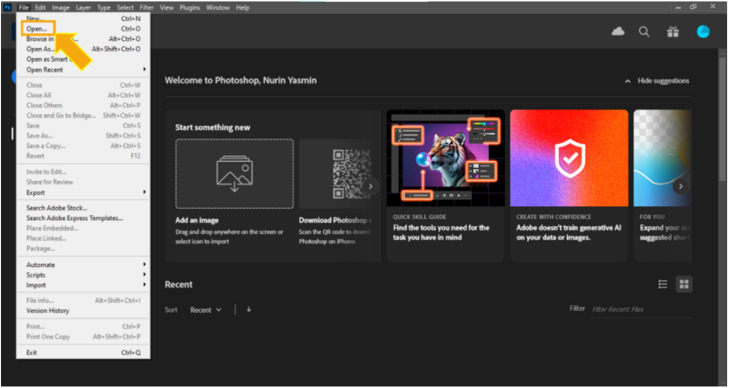
The Yellow arrow indicates the “Open” option under the File menu in Adobe Photoshop.
3. Select an Image of the plaque that will be quantified; the selected photo will appear in the windowpane (Fig. 12).

**Fig. 12:**
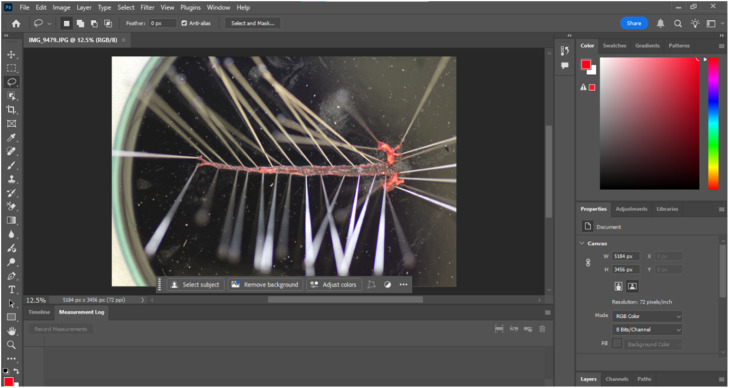
The selected image appears in the windowpane.
4. Go to Image> Analysis> Set Measurement Scale> Default; 1 pixel= 1.000 pixels (Fig. 13).

**Fig. 13:**
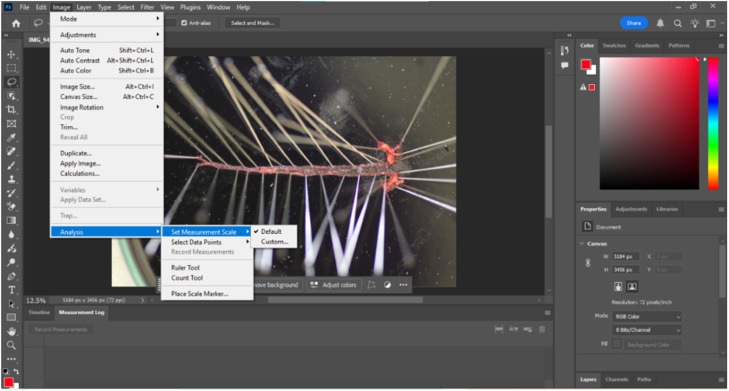
Setting of the analysis.
5. Go to Window.
6. Click the Measurement Log Panel; the panel will appear at the bottom of the window (Fig. 14).

**Fig. 14:**
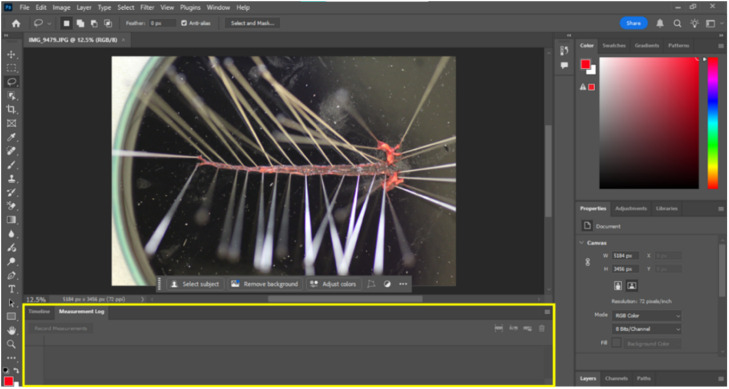
The yellow box indicates the measurement log panel.
7. Select the Polygonal Lasso Tool to begin measurement (Fig. 15).

**Fig. 15:**
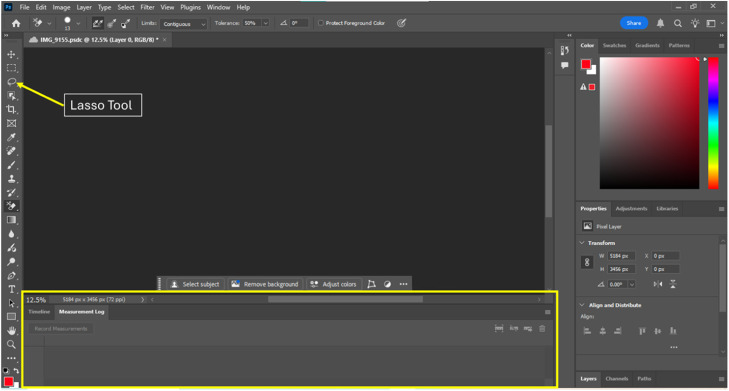
The yellow arrow indicates the Polygonal Lasso Tool options.
8. Using the Polygoonal Lasso Tool to begin the measurement, outline the entire area of the aorta in one continuous move. Ensure that the outline fully covers the whole aortic area. It might require some practice at first (Fig. 16).

**Fig. 16:**
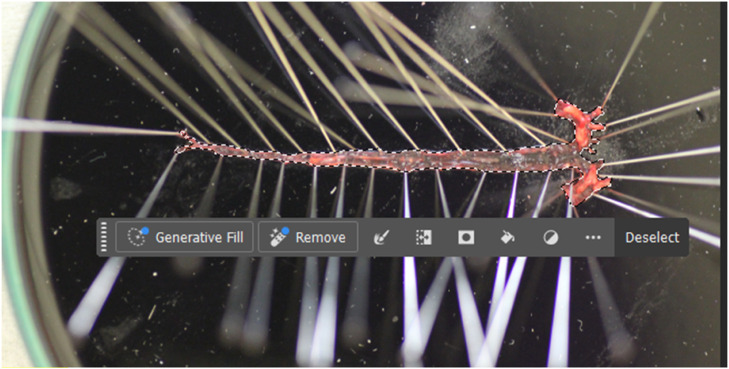
Dashed line indicates the outline of the entire aorta using the Polygonal Lasso Tool.
9. Click Record Measurement (Fig. 17).

**Fig. 17:**
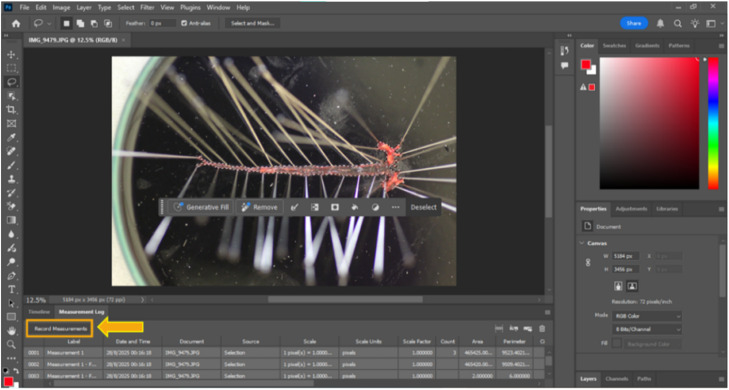
The yellow arrow indicates the Record Measurement Button.
10. Once the entire aorta measurement is recorded, outline all the visible red-stained plaque lesions indicated in ORO staining. The Record Measurement button needs to be clicked after each measurement. Click View > Zoom In as needed when measuring. If continuous measurement is preferred, hold the “Shift” key and start to outline each plaque. Release the “Shift” key once all of the measurements have been completed, and click “Record Measurement” (Fig. 18)

**Fig. 18:**
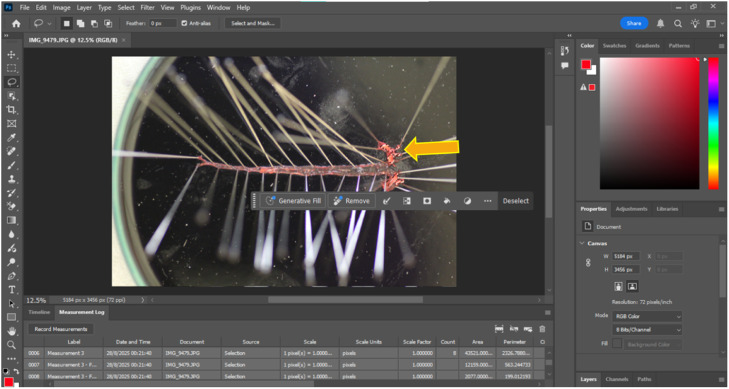
The yellow arrow indicates the dashed outline representing one red-stained plaque area at the aortic arch that has been delineated for measurement.
11. Export all data by saving the file in txt format, then select both “Select All Measurement” (yellow box) and “Export Measurement” (red box) (Fig. 19).

**Fig. 19:**
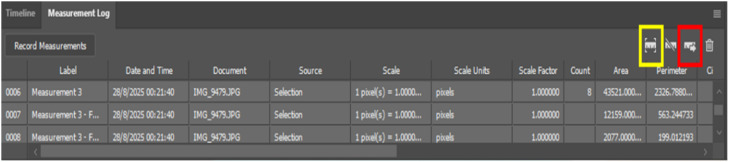
The yellow box indicates the “Select All Measurement” button, and the red box indicates the “Export Measurement” button.
12. Open Microsoft Excel and access the saved .txt file from the File menu.
13. To open as an “Excel” file, click on the .txt file, and rename it to .csv (Fig. 20).

**Fig. 20:**
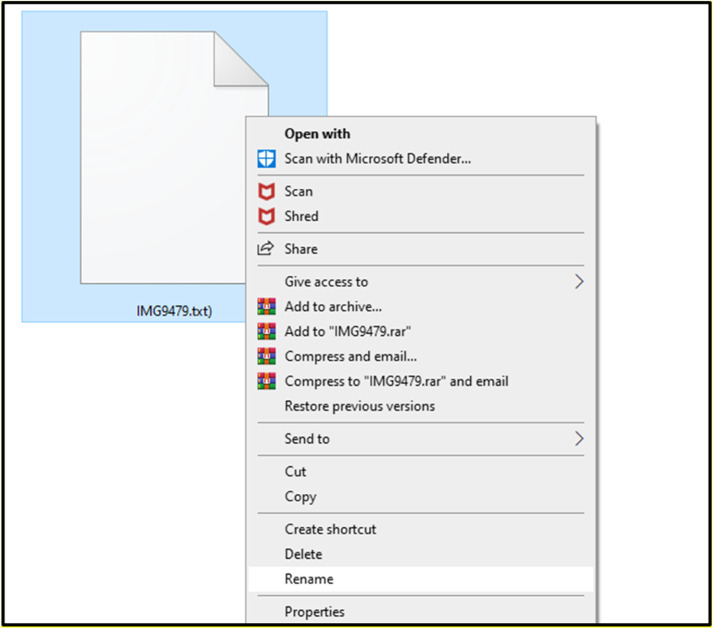
Renaming the exported measurement file from “.txt” to “.csv” format to enable opening in Microsoft Excel.
14. Once replaced .txt to .csv format, the file was able to be accessed using Excel. Click the file to start measuring the raw data.
15. Once the raw data were accessed in an Excel file (Fig. 21), each data point was re-labelled, and unusable data were deleted or kept in another tab. Data cleanup was executed carefully to avoid removing important data. In Fig. 21, the data indicated in the yellow box represent the total measured area of the aorta, while the data in the red box correspond to the red-stained plaque areas obtained from the *en face* image. In each dataset, the first row indicates the cumulative data point count; for example, the value “3” denotes that three individual outlines were used to calculate the total aortic area. The subsequent three rows correspond to the individual outlines of the measured regions. In this example, the total aortic area is 465,425 pixels, and the total plaque area is 43,521 pixels, representing the cumulative area of all red-stained plaque lesions measured within the whole aorta.16. Each datapoint was then relabelled properly. Data were selected and calculated using the formula: Total Percentage of Plaque Area = Area of Artery containing plaque / Total Area of artery x 100% (Fig. 22).

**Fig. 21:**
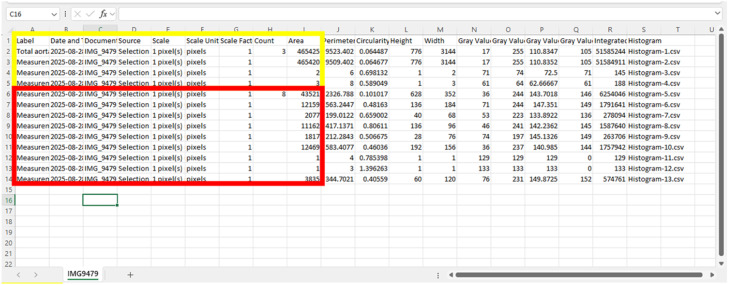
Excel sheet showing raw measurement data. The yellow-box section indicates the total aortic area, and the red-box section represents the red-stained plaque areas. The first row shows the cumulative data point count, and the subsequent rows represent individual outlines of the measured areas (values in pixels, based on the default scale setting of 1 pixel = 1.000 pixels).

**Fig. 22:**
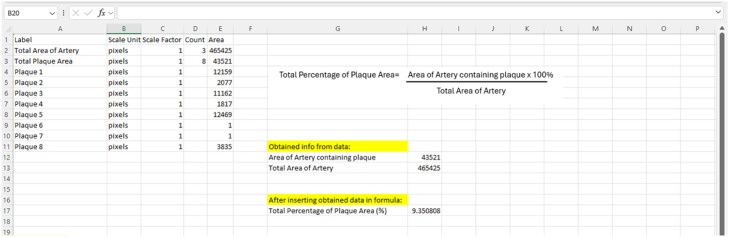
Excel sheet showing calculation of total plaque area percentage using measured data and applied formula.

### Post-staining sample storage

**Fig. 23:**
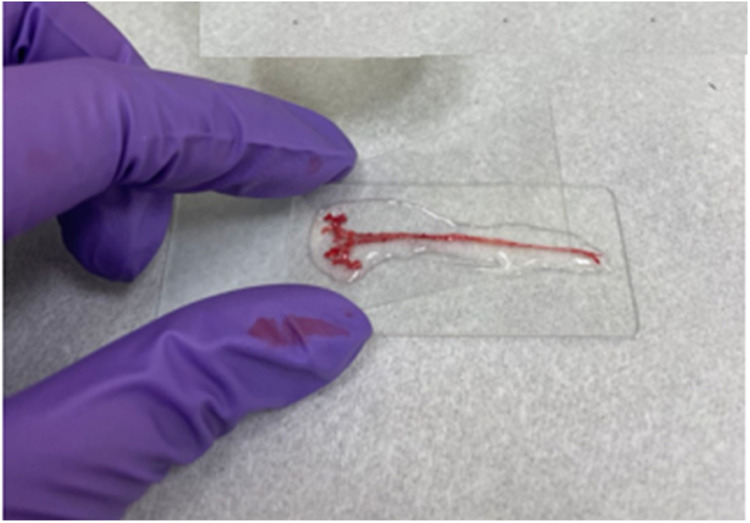
ORO-stained aorta mounted on a microscope glass slide by using OCT and sealed with a cover slip.

The aorta was removed from the Black Sylgaard Petri Dish (DMT) and mounted on standard glass microscope slides (75 × 25 × 1 mm) and covered with No 1.5 coverslips (0.17 mm thickness) (Fig. 23). Mounting medium used was Optimum Cutting Temperature Compound (OCT) (Sigma Aldrich, USA).

## Method validation

A modified *en face* ORO staining protocol applied to a 70-week-old ApoE knockout mouse in this study produced a clear and uniform staining pattern in the entire aorta from the arch to the iliac bifurcation. The luminal surface could be completely observed after the specimen was flattened, and the individual lipid-rich plaques appeared as bright red objects against a pale background.

The plaque burden was found to be highest at the aortic arch and its branches, and decreased distally down the aorta and into the iliac sections. This localization is consistent with the hemodynamic locations of plaque susceptibility in ApoE-deficient murine models described earlier [14].

The use of Adobe Photoshop-based digital quantification allows the objective measurements of plaque area in relation to the total aortic surface. The Polygonal Lasso tool was used to outline plaque-positive areas and plaque lesion percentage was obtained through the stated formula. The employment of a typical digital camera and Adobe Photoshop software provided an economic, easy alternative to imaging systems relying on a microscope and morphometric special instrumentation.

Unlike the conventional cross-sectional histology, this *en face* approach provided a more comprehensive spatial overview of plaque distribution while preserving tissue integrity [8]. The practicality and simplicity of this workflow of *en face* ORO staining in the current paper, along with its ability to be visualized step by step via digital recording and reproduced, make it especially appropriate for large-scale or longitudinal studies in preclinical atherosclerosis research.

Regardless of the number of established *en-face* ORO procedures that have been reported in the literature [1,2,[8], [9], [10], [11], [12], [13]], this study distinguishes itself from previous studies by displaying a step-by-step photographic documentation starting from aortic staining procedures down to plaque quantification procedures. Most previous studies did not thoroughly demonstrate the step-by-step plaque quantification procedures.

## Limitations

ORO staining is a widely used method for visualizing lipid-laden atherosclerotic plaques, especially when en-face visualization of the aorta is required; however, it has various methodological limitations. The stain specifically stains neutral lipids, cholesterol esters and triglycerides, but not other salient plaque components, including inflammatory cells, mineral deposits, and fibrous tissue [15].

The protocol requires fresh or frozen specimens, as lipids are removed in the process of paraffin embedding, which limits the compatibility of the method with specific histological protocols [16]. The quantitative evaluation is limited to two-dimensional surface-area measures, which excludes the information on plaque depth or compositional heterogeneity.

Additionally, background staining may be caused by residual fat or inadequate rinsing. The procedure also relies on uniform tissue dissection and accurate image thresholding, which can result in variability between operators or laboratories. Although the red chromogen from the reagent can be used to visualize lesions, it is relevant to note that this technique also stains neutral lipids in the adventitial adipose tissue. This can be mistakenly recognized as a plaque lesion and, therefore, careful elimination of adventitial fat is required [17]. Importantly, certain lesions can be delicate and fragile and may be easily displaced during the staining procedure.

Apart from this, selecting an appropriate mouse model and supplier is crucial to ensure optimal visualization in *en face* ORO analysis, where plaque formation must be sufficiently evident for preparing a protocol manuscript. In this study, a 70-week-old ApoE-knockout mouse was obtained from Taconic Biosciences, a well-established supplier of laboratory animals recognized for its consistent genetic background and rigorous quality control.

### Ethics statements

All procedures in this study were approved and conducted in accordance with Animal Ethics guidelines from the UiTM Research Ethics Committee, UiTM CARE: 426/2023.

### CRediT author statement

NYMK performed the experimental work, staining procedures, imaging, and data quantification. NA conceptualized and designed the study, provided technical expertise in the methodological optimization, and supervised the overall project. VB and SAM provided methodological input and critical manuscript review. NN assisted with lab setting and figure preparation. NA and NYMK drafted the manuscript. All authors discussed the results, reviewed the final version, and approved the manuscript for submission.

### Declaration of generative AI and AI-assisted technologies in the manuscript preparation process

During the preparation of this work, the authors used ChatGPT (https://chatgpt.com/) and Grammarly (https://app.grammarly.com/) to enhance its readability and accuracy. ChatGPT was used to improve the readability and clarity of the text, while Grammarly was used to correct the spelling errors. The authors reviewed and edited the output as needed and take full responsibility for the content of the published article.

## Declaration of competing interest

The authors declare that they have no known competing financial interests or personal relationships that could have appeared to influence the work reported in this paper.

## Data Availability

Data will be made available on request.
